# Quantification of myocardial perfusion by cardiovascular magnetic resonance

**DOI:** 10.1186/1532-429X-12-57

**Published:** 2010-10-08

**Authors:** Michael Jerosch-Herold

**Affiliations:** 1 Brigham & Women's Hospital, Boston, MA 02115, USA

## Abstract

The potential of contrast-enhanced cardiovascular magnetic resonance (CMR) for a quantitative assessment of myocardial perfusion has been explored for more than a decade now, with encouraging results from comparisons with accepted "gold standards", such as microspheres used in the physiology laboratory. This has generated an increasing interest in the requirements and methodological approaches for the non-invasive quantification of myocardial blood flow by CMR. This review provides a synopsis of the current status of the field, and introduces the reader to the technical aspects of perfusion quantification by CMR. The field has reached a stage, where quantification of myocardial perfusion is no longer a claim exclusive to nuclear imaging techniques. CMR may in fact offer important advantages like the absence of ionizing radiation, high spatial resolution, and an unmatched versatility to combine the interrogation of the perfusion status with a comprehensive tissue characterization. Further progress will depend on successful dissemination of the techniques for perfusion quantification among the CMR community.

## Introduction

The title of this review, "perfusion quantification" refers to the approaches used with cardiovascular magnetic resonance (CMR) to assess or measure myocardial blood flow from the contrast enhancement observed during the first pass of a contrast agent bolus. This technique is often referred to by the name of "first pass" imaging, because the first pass of a contrast agent represents the phase of contrast enhancement most sensitive to changes in blood flow, be it from disease, pharmacological intervention, or exercise. Another approach, not requiring exogenous contrast, and referred to as arterial spin labeling, can also be employed to assess myocardial blood flow[1]. Although a promising technique that is not burdened by concerns about contrast administration in patients with poor renal function, it still remains largely confined to experimental studies, challenged by poor contrast-to-noise, and arguably only practically relevant for cardiac studies at 3 Tesla, or higher field strengths. Another promising technique for assessing myocardial perfusion is based on blood-oxygen-level dependent (BOLD) myocardial imaging[2,3]. This type of endogeneous contrast mechanism is dependent on a combination of factors such as blood flow, but also oxygen extraction fraction, with the latter normally declining with increasing myocardial blood flow[4]. BOLD imaging does not provide as direct an assessment of myocardial blood flow, as contrast-enhanced perfusion imaging. This review will focus on the quantification of myocardial perfusion by the use of contrast-enhanced techniques, which in the future may include novel contrast agents such as hyper-polarized media[5], but at the present time is mostly performed by the administration of gadolinium chelates as contrast agent. For the purpose of this review article, we define the quantification of myocardial perfusion as methods or approaches to determine the tissue blood flow in the heart muscle. A recent comprehensive review with considerable detail on the technical aspects of myocardial perfusion imaging can be found in[6].

The quest to quantify myocardial perfusion has been largely motivated by the desire to obtain quantitative, observer-independent, and reproducible measures of the myocardial perfusion status. Whether a quantitative approach improves the accuracy of myocardial perfusion imaging, such as for the detection of coronary artery disease, remains controversial, with circumstantial evidence pointing to the benefits of a quantitative analysis, versus a qualitative interpretation in cases of multi-vessel disease. But in the research realm, there is by now a long line of evidence proving that the measurement of myocardial perfusion leads to new insights in coronary physiology and the etiology of cardiac diseases.

## CMR Acquisition Methods

In the context of perfusion quantification, there are two aspects of myocardial perfusion studies that warrant some discussion about the methods for dynamic image acquisition. First of all, one assumes that the observed contrast enhancement is proportional to the change of contrast concentration in the tissue. For example, if one were to use a pulse sequence giving mixed T1/T2* contrast, with T1 effects prevailing at lower contrast concentrations, and T2* effects dominating at higher concentrations, then this would give rise to signal increases at lower concentrations, which would be confounded by signal loss at higher concentrations. It is preferable to have a sequence technique that makes one contrast mechanism (e.g. T1) prevail. T1-weighted imaging is predominantly used for myocardial perfusions studies, not the least because the vascular volume and also the distribution volume of Gd-chelates is sufficiently large to yield appreciable signal intensity changes in myocardial tissue. The short echo times inherent in weighting the signal towards T1 are advantageous for minimizing the effects of myocardial motion and flow, as one would otherwise have increasing signal attenuation due to motion and flow when the echo time (TE) becomes longer.

A second aspect relates to the nature of contrast enhancement in the blood pool of the ventricular cavity or the proximal aorta. Intuitively, it is obvious that myocardial contrast enhancement is driven by arterial enhancement. In fact, we will see that the myocardial contrast enhancement can be considered as a linear response to the arterial contrast enhancement, which implies that myocardial contrast enhancement can never proceed at a rate faster than for the arterial contrast enhancement. For this reason it is important to measure the contrast enhancement in the blood pool as reference for the analysis of the myocardial contrast enhancement. Ideally, the arterial contrast enhancement would be measured as close as possible to the myocardial region under consideration, but in practice one can only expect to measure enhancement in the blood pool at a location in the left ventricle, or in the proximal aorta. Either way, the pulse sequence should not be overly sensitive to blood flow in the great vessels or the ventricular cavities, nor should it suppress the signal from flowing blood. For example, pulse sequences with long echo-trains after each radio-frequency excitation would suppress signal from blood flow in the ventricular cavities and should be avoided for quantitative perfusion studies.

The contrast enhancement in the blood pool has a non-linear or sub-linear dependence on contrast concentration, with the nonlinearity, or contrast-enhancement saturation, becoming more pronounced as contrast concentration increases [7,8]. Eventually, the signal intensity would even start to decrease above a certain contrast level, due to T2* effects. Signal saturation effects need to be avoided or corrected, if one wants to accurately measure the arterial input of contrast. Methods for quantifying myocardial perfusion use the arterial input as reference. Any systematic underestimate of arterial contrast enhancement results in an overestimate of myocardial perfusion, i.e. relative to the arterial contrast enhancement, myocardial contrast enhancement appears to be higher than is truly the case, and assuming negligible myocardial signal saturation.

Possible solutions to avoid arterial signal saturation are: a) the use of lower contrast dosages, which avoid the saturation effect, albeit at the price of reduced contrast-to-noise in the myocardium; b) employing dual contrast sequences[8,9]; c) using a dual contrast bolus protocol[10], and d) correcting retrospectively for signal saturation, e.g. by using calibration curves[11,12] (see section below on Signal Intensity and Contrast Concentration). Briefly, the dual-contrast sequences include low-resolution dynamic imaging of the enhancement in the ventricular cavity, in addition to high(er) resolution imaging of myocardial enhancement. With a 2D dual-contrast sequence one images several slices (e.g. in short axis view) during a heart beat to capture the myocardial contrast enhancement with strong T1 weighting, and during the same heart beat also acquires a low resolution image of the contrast enhancement in the ventricular cavity, with lower T1-weighting than for the myocardium, to avoid saturation at higher contrast concentrations. The low resolution blood pool image has a low T1 weighting, because fewer phase-encodings are carried out between magnetization preparation and read-out of central phase-encodings, resulting in a shorter delay after inversion (TI) or saturation. The short delay, and resulting low-T1 weighting, improves the linearity of the signal-intensity vs. R1 relationship at higher contrast concentrations.

The dual bolus approach involves giving a low dosage contrast bolus to characterize the arterial input of contrast, followed by a higher dosage bolus to image the myocardial contrast enhancement. The two bolus dosages are in a pre-determined ratio (e.g. 1:10) that is then used to scale the arterial input function (AIF) from the low-dosage bolus to analyze the myocardial contrast enhancement with the rescaled and time-shifted AIF.

A further important aspect is the administration of contrast: If the contrast is injected slowly, then the observed myocardial contrast enhancement is bottlenecked by the slow arterial enhancement, and myocardial enhancement becomes relatively insensitive to blood flow. To achieve good sensitivity to myocardial blood flow, the contrast agent should be injected as a bolus, at a rate somewhere between 3-5 ml/s[13], in particular for measurements of the hyperemic response. In other words, as the blood flow increases, the requirement for a bolus injection become more demanding, while for resting flows, injection rates as low as 1 ml/s are acceptable. There is no stringent reason why the injection rates for rest and hyperemic flow measurements need to be the same, although in practice this is generally the case.

The signal-intensity-based analysis of myocardial perfusion can be confounded by spatial variation of the coil sensitivity profiles, e.g. with phased-array receive coils, and also by B_1 _inhomogeneity effects, e.g. due to dielectric effects, in particular at magnetic field strengths of 3 Tesla or higher. Body RF coils, used almost exclusively for RF excitation, are designed to achieve excellent B_1 _homogeneity, albeit under assumed ideal conditions. B_1 _inhomogeneity over a region with the dimensions of the heart can be brought about by two conditions: a) the electromagnetic wavelength of the RF excitation can approach the field of view dimensions at higher field strengths, and in tissue the wavelength can be even shorter, because tissue has a comparatively high relative dielectric constant or permittivity (e.g. ε_r_~70 at 100 MHz for muscle tissue); and b) the dielectric properties within the anatomical region being imaged can be highly heterogeneous, thereby giving rise to dielectric resonances, a form of constructive B_1 _interference effects that can be set up in a dielectric "cavity". B_1 _inhomogeneity can contribute to signal intensity variation over the heart, but equally important, it also causes unintended changes for the magnetization preparation. For the latter, it is useful to realize that a magnetization preparation with a nominal inversion pulse that deviates from the ideal 180° flip angle can be confounded with a more rapid relaxation recovery, and similar observations apply to saturation pulses. In other words, in the presence of B_1 _inhomogeneity, the signal intensity variations can mimic perfusion defects. Currently, the most common solution for myocardial perfusion imaging at ≥ 3T, is the use of adiabatic, i.e. B_1_-insensitive, RF inversion or saturation pulses, or composite pulses. Adiabatic pulses increase the SAR burden, because the B_1 _amplitude intrinsically has to be considerably higher than normal (to meet the adiabaticity condition), and the pulses also have 5 to 10-fold longer durations, both of which increase the deposited RF energy (proportional to *B_1_^2^*·*Δt*, over a time interval *Δt *of the pulse, during which B_1 _is approximately constant). Composite pulses represent a practical compromise with lower SAR burden than adiabatic pulses, but they still compensate for B_1 _inhomogeneity or flip-angle variation[14].

The spatial variation of receive coil sensitivity, although also a potential source of misinterpretation of contrast enhancement, can be corrected for in practice by mapping out, or estimating the coil sensitivy profile(s). Mapping of the coil sensitivity profiles is a prerequisite for reconstruction of images acquired with parallel imaging acceleration, which intrinsically also takes care of the coil-sensitivity variation in the reconstructed images. Otherwise, coil sensitivity has to be estimated from one of the pre-contrast images, and preferably images acquired with proton-density weighting, and insensitive to in-flow in the ventricular cavity ("bright blood effect"). Recent implementations of myocardial perfusion sequences include such proton-density acquisitions as the first 1-2 images in a dynamic imaging scan, by leaving out the magnetization preparation, and using a small flip-angle for the image read-out[15]. To reduce the impact of noise, the data of different myocardial segments within one slice can be fitted to a sinusoidal function[15]. A sinusoidal variation would be a first-order approximation, assuming a locally linear spatial variation of the signal intensity, and a circular short axis cross-section of the left ventricle (LV). A common way to correct for intrinsic, spatial signal intensity variations is to divide the myocardial signal by its pre-contrast value. This corrects for variations for the signal within the myocardium, due to receive-coil inhomogeneity, but for the blood pool a similar correction is only feasible if pre-contrast-signal is not enhanced by flow, and predominantly proton-density weighted. It is also implicitly assumed that the proton density of myocardium and blood are about the same.

## Pulse Sequence Techniques and Perfusion Quantification

Techniques for image acquisition in myocardial perfusion studies have spanned, in approximate order of image acquisition speed, the range from (spoiled) gradient echo imaging with cartesian, or radial[16] k-space trajectories, through gradient echo imaging with steady state free precession[17,18], to echo-planar[19-22], and spiral techniques[23]. The spoiled gradient-echo technique is the slowest, but least susceptible to artifacts from off-resonance shifts (i.e. field inhomogeneities), T2* and susceptibility effects, and flow and motion. Compared to an acquisition with steady state free precession, the spoiled gradient echo technique suffers from nearly two-times lower signal-to-noise[17], but steady-state free precession techniques are currently not a viable option for perfusion imaging at 3 Tesla or higher field strengths, because of image artifacts. Echo-planar techniques are mostly used in a hybrid form, where the echo-train length is limited to approximately 3-6 echoes, depending on field strength and T2*. Similarly, the spiral technique is not used in a single-shot read-out mode for contrast-enhanced myocardial perfusion imaging, but instead interleaved spirals are acquired to reduce the effective T2* weighting of the signal. Whether a particular technique is suitable for quantitative myocardial perfusion imaging depends on details of its implementation on any particular scanner platform, field strength, and contrast agent. It is conceivable that a particular technique may work with a standard gadolinium-chelate, but that with a iron-based contrast agent, T2* effects cause too much susceptibility artifacts.

## Signal Intensity and Contrast Concentration

The quantitative assessment of myocardial perfusion relies on measuring the rate at which tracer (i.e. contrast agent in the case of "first pass" perfusion studies) arrives in a tissue region of interest. The tracer concentration in tissue is detected indirectly by the T1 effect of the contrast on the 1H signal. T1 in a homogeneous voxel (i.e. without compartmentalization) is directly proportional to the contrast concentration, and the proportionality constant is the T1-relaxivity of the contrast agent (*r_1_*):

(1) $$R_{1}=R_{1n}+r_{1}\cdot [Gd],$$

where R_1n _denotes the native T_1 _relaxation rate constant (i.e. in the absence of any contrast agent) and *[Gd]*, the concentration of Gd contrast in the voxel. The relaxivity of most Gd-chelate agents is unchanged as it transitions from blood into tissue, with albumin-binding agents being one notable exception.

Any pulse sequence yields a finite dynamic range for signal changes when contrast is introduced, with a theoretical upper bound set by the proton density. We give here a specific example for a spoiled gradient echo sequence, with saturation recovery magnetization preparation, and linear ordering of the phase-encodings. After N/2 phase-encoding steps (i.e. the number of phase-encodings to reach the central k-space line) the signal intensity is given by[24]:

(2) $$S_{N/2}=S_{0}\left[(1-\exp[-R_{1}TD])\cdot a^{N/2-1}+(1-E)\frac{1-a^{N/2-1}}{1-a}\right],$$

where *S_0 _*is the equilibrium signal, *TR *the repetition time per phase encoding, *R1 *the T1 relaxation rate (*R1 = 1/T1*), *TD*, the delay between magnetization preparation and start of FLASH read-out, *E = exp(-R1·TR)*, and *a = E·cos(α)*, with α denoting the flip angle. The signal intensity of the central-k-space echoes determines the overall contrast characteristics of an image, although contrast enhancement in smaller structures, is also weighted by the T1-weighting of higher-frequency k-space echoes. The effects of the modulation of the phase-encoding amplitudes by an inversion, or saturation recovery have not been systematically investigated, but could impact negatively on the quantification of blood flow, e.g. in thinned LV walls.

**Figure 1:**
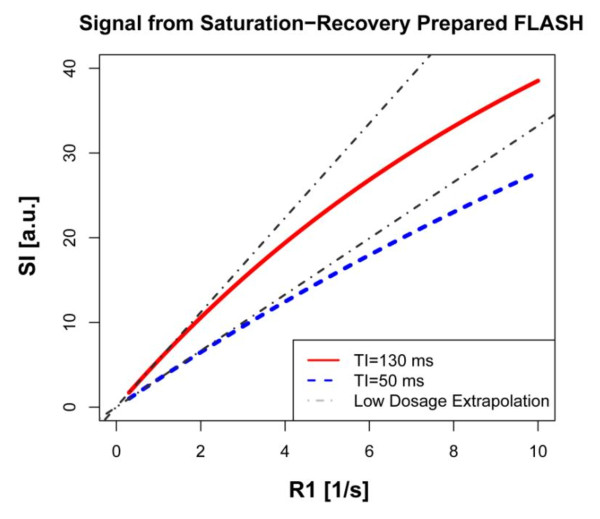
**Function of the relaxation R1**. The signal intensity of the central k-space lines, which determines the overall image contrast, was calculated for a rapid gradient echo sequence (TR = 2 ms, flip angle = 15°) with magnetization preparation and linear phase-encoding order as a function of R1. The red line is for the case where the temporal separation (TI) between saturation pulse and the central k-space line equals 130 ms, corresponding to a total of 128 phase encoding steps. The blue curve corresponds to a low-resolution image with 45 phase-encoding steps. The signal intensity initially shows a linear dependence on R1, which starts to show a convex shape for higher R1 values. This deviation from the linear dependence is referred to as signal saturation. The initial linear dependence of SI on R1 was extrapolated to larger R1 values, and represents the ideal case for a quantitative perfusion analysis, where signal intensity in blood is linearly proportional to contrast concentration. For the shorter TI, the linear regime extends to higher R1 values, than with a longer TI. A short TI setting is therefore preferable to avoid signal saturation (e.g. in the blood pool).

The expression in [2], plotted in Figure 1 as function of the relaxation R1, shows a linear dependence of the signal intensity at low R1 values (long T1's), and then takes on a convex shape at higher R1 values. The deviation from the linear extrapolation for short R1's is most apparent with longer times between magnetization preparation (saturation pulse) and read-out of the central k-space line. Ideally, one would want the signal intensity to have a linear dependence on R1, as shown by the grey, dashed-line extrapolations in Figure 1. To first approximation, this can be achieved by using lower contrast dosages (< 0.05 mmol/kg at 1.5 T with Gd-DTPA). Any deviation from a linear dependence of the signal on R1, or tracer concentration, will be most apparent in the blood pool of the LV cavity or the aorta, where one measures the arterial input: downstream from the arterial input the contrast bolus becomes more dispersed, which means that the peak contrast concentration is lower. The fact that the observed peak signal intensity in tissue is much lower than in the blood pool does not imply that there is no signal saturation in the tissue. It simply reflects the fact that the distribution volume in tissue is < ~30%, but the signal intensity contribution from the vascular space could very well suffer from saturation effects. The attention for correction of saturation effects has nevertheless focused primarily on the signal in the blood pool, in good part because it is readily discernible when the peak of the bolus is flattened by saturation, and also because there exist approaches to correct for saturation. Signal saturation for the vascular component of the signal in tissue has not been addressed much in the literature, arguably because it represents a more complex challenge.

With pre-contrast T1 measurements, knowledge of the sequence parameters, and numerical simulation, it is possible to correct the signal saturation, by generating calibration curves to replace the measured signal intensity, or percent contrast enhancement, by their corresponding values in the absence of signal saturation[11,12]. Alternatively, one can measure the signal intensity calibration curves, but this turns into a more tedious approach, and with a change of sequence parameters it may be necessary to regenerate a calibration curve. Details on methods for saturation correction can be found in[11,12]. If the saturation effect is neglected, then the contrast enhancement in the myocardium will appear to be larger *relative *to the arterial contrast enhancement, and myocardial perfusion is overestimated. The overestimate of blood flow can be almost proportional to the peak saturation effect, i.e. a 30% reduction of the peak signal intensity of the arterial input due to saturation can cause an overestimate of myocardial blood flow of similar relative magnitude.

## Signal Intensity Artifacts

Although it may go without saying that signal intensity curves for myocardial regions of interest should represent only the underlying tissue properties, this may be difficult to achieve for the endocardial layer, because the regional signal intensity average may include an admixture from the ventricular blood pool. Several factors can contribute to this, all too often, subtle artifact: the endocardial border definition may be poor, which can lead to the inadvertent inclusion of some blood pool region, or some voxels may only partially be filled by myocardial tissue over the full slice thickness, which generally exceeds by a factor of 4-5 the in-plane voxel dimensions ("partial volume effect"). These partial-volume effects are also referred to as "spillover" from the blood pool, a term particularly prevalent in nuclear medicine. In nuclear medicine spillover correction was incorporated by adding to any model for the myocardial contrast enhancement, a "spillover" term, which essentially amounts to a scaled (and time-shifted) arterial input function, with the scaling factor (and time shift) as variable parameter(s)[25]. Such an approach has also been tested successfully in MRI studies of myocardial perfusion[26]. By virtue of the higher spatial resolution of MRI, it may be preferable in the future to further optimize the image acquisition to avoid spillover, rather than trying to estimate any signal admixture from the blood pool, in addition to the tissue perfusion parameters.

A persistently vexing problem in myocardial perfusion studies has been the appearance of dark rim artifacts at the endocardial border, when a contrast bolus first enters the ventricular cavity[27,28]. Several potential mechanisms have been proposed to explain this phenomenon, with arguably the two most likely causes being, the effect of Gibbs ringing near a sharp and large signal intensity jump, and magnetic susceptibility effects, which cause intra-voxel dephasing at the endocardial border. At the stage of post-processing, the choices to deal with this artifact are limited. One can avoid the region with the dark rim when the endocardial contour is drawn, by pulling the contour back towards the epicardial border, but then runs the risk of missing a perfusion defect limited to the subendocardial layer, which is the layer most vulnerable to ischemia. Alternatively, one can leave out the data points in the signal intensity curve where the signal intensity drops significantly below the pre-contrast baseline level. This is clearly also an unsatisfactory solution, and generally requires that the signal intensity curve is constrained by a model to estimate blood flow. With a semi-quantitative, model-independent analysis, such as determination of the signal-intensity up-slope, the missing data-points may render it impossible to estimate the semi-quantitative perfusion parameter.

## Units of Perfusion Measurements

Measurements of arterial and myocardial contrast enhancement do not require any calibration in terms of absolute units for contrast enhancement. It is sufficient that they are measured on the *same linear *scale, irrespective of whether that scale has absolute or arbitrary units. The time points for signal intensity readings need to be recorded in absolute units, for example seconds, relative to a common reference for all images, such as the start of the image acquisition. One can then quantify the contrast enhancement as fraction of the arterial contrast enhancement (ml of arterial input per ml of tissue), and per unit of time. While the arterial contrast enhancement is typically measured per unit volume of blood, tissue blood flow is quoted in mL of arterial input per gram of tissue, which requires that the unit volume of myocardial tissue be converted into its mass equivalent, using the specific gravity of myocardial tissue, which averages 1.05 g per ml of tissue. Units of ml/min per g of tissue follow naturally from the procedures for microsphere tissue blood flow measurements, where the deposition of tracer is measured per g of tissue sample. The use of microspheres as reference standard made the units of ml/min per g of tissue also a natural choice for estimates of myocardial blood flow with external detection by an imaging device (MRI, CT, PET, etc.).

## Semi-Quantitative Perfusion Measures

**Figure 2:**
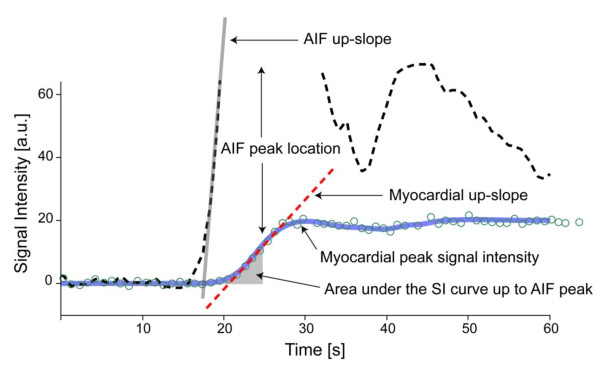
**The characteristics of myocardial contrast enhancement**. The characteristics of myocardial contrast enhancement have been assessed in the literature using several parameters, as illustrated in this example, showing the signal intensity changes in the LV blood pool (arterial input function ℵ AIF), and in an anterior segment of the left ventricle (green circles). The blue line shows the fit to the measured data with a two-space distributed model. The dashed red line is commonly referred to as the up-slope parameter, and gives the initial rate of contrast enhancement. It is often normalized by the up-slope of the AIF, as an empirical correction factor to account for hemodynamic changes between rest and stress. The area under the tissue curve (gray shaded area), up to the location in time where the peak of the AIF is observed, has been proposed as an alternative parameter to assess perfusion and the perfusion reserve. The myocardial peak signal intensity during the first pass of the contrast bolus in the LV is arguably the least flow-sensitive parameter, and therefore only used to assess contrast-to-noise, but not changes or differences in myocardial perfusion.

Before discussing approaches for quantifying myocardial blood flow from CMR perfusion studies, brief mention should be made of semi-quantitative perfusion measures, which form the basis of perfusion reserve indices. One such parameter is the so-called "up-slope"[29], which refers to the slope of the signal-intensity curve during the early phase of contrast enhancement. More recently the area under the myocardial signal curve, up to the time where the first pass peak is observed in the blood, was used to estimate the perfusion reserve, and validated against microsphere measurements[30]. Figure 2 illustrates parameters derived from myocardial signal intensity curves, which have been used as semi-quantitative markers of tissue perfusion. When measured from CMR "first pass" studies, the up-slope parameter has an approximately linear dependence on blood flow. It can be used to gauge the relative variation of blood flow within the LV wall during a given hemodynamic state. But like nearly all parameters derived from signal intensity curves, it depends on the shape of the arterial input, and therefore also on the hemodynamic conditions. When the up-slope parameter was initially proposed to assess myocardial perfusion, investigators normalized it by the up-slope of the signal-intensity in the LV blood pool[29]. A slightly different form of normalization can be defined, based on the central volume principle and a linear approximation for the initial arterial input[31]. These empirical adjustments for differences of the arterial input between hemodynamic states were used to define a perfusion index, which could be calculated for rest and stress, and the ratio of the stress value, divided by the rest value was used as a perfusion reserve index. The rationale for forming such ratios goes back to the concept of the coronary flow reserve (CFR), which corresponds to the ratio of coronary flows at stress and baseline[32].

One advantage of such a perfusion index is the relative simplicity of its estimation from the signal intensity curves. For each perfusion parameter, and with some form of adjustment for hemodynamic conditions, one can in principle define a perfusion reserve index. While these perfusion reserve indices may be proportional to the coronary flow reserve over some limited range of CFR, or the perfusion reserve obtained from flows measured by the microsphere method, they generally each have specific thresholds to distinguish a hemodynamic significant stenosis from a coronary artery without flow-limiting lesions. The semi-quantitative perfusion indices cannot be compared in magnitude directly to the coronary flow reserve ratio measured in the catheterization laboratory. A further drawback of any ratio is the potentially confounding effect of the quantity in the denominator, which purportedly serves the role of "normalization"[33]. An example is the measurement of the perfusion reserve in hypertensive patients, where rest perfusion, which increases in proportion to the rate-pressure product, may be abnormally high. Whether the perfusion reserve in a hypertensive patient is reduced because of elevated resting blood flow, or an impaired hyperemic response, or a combination of both cannot be determined from a ratio, unless the quantities in the numerator and denominator can be assessed independently. The latter is only feasible with absolute myocardial blood flows.

## Model-based Quantification of Myocardial Blood Flow

The approaches which can be used for quantifying myocardial blood flow from the observed contrast enhancement can be broadly divided into two categories, which we label here as model-based, and model-independent. For model-based approaches one specifies the functional spaces in myocardial tissue, how tracer moves through these spaces, and how it traverses permeable barriers between spaces. A considerable degree of simplification is necessary to arrive at models that can be used for numerical calculations and simulations. As commonly used MR contrast agents, such as Gd-DTPA, are excluded from the intracellular space one can consider a simplified model comprising only the vascular and interstitial spaces. Such a "two-compartment" or "two-space" model[34] can be used to describe the contrast enhancement, i.e. the change of signal above its pre-contrast-injection baseline level. In other words, the background signal, comprising also a contribution from the intracellular space is subtracted before model-based-analysis of the contrast enhancement. Such a baseline correction can also be appropriate for analyzing the "first pass" enhancement after a previous contrast injection, if the background signal has reached an equilibrium level. Generally, one should wait at least 10 minutes between first pass imaging scans. In our experience, T1 in the blood pool at approximately 10 minutes after contrast injection reaches approximately 60% of its pre-contrast level. This corresponds to approximately 1/10^th ^to 1/20^th ^of the peak contrast-enhancement observed with a 0.03-0.04 mmol/kg bolus of Gd-DTPA, and therefore amounts to approximately 5-10%, or less, of the peak contrast enhancement observed during a second injection, an arguably acceptable level for the background signal increase in the blood pool. In the myocardium, the signal intensity level at 10 minutes after contrast injection is only ~10-20% higher than before contrast injection, which is a small fraction of the peak myocardial contrast enhancement during a first pass. After 10 minutes or longer, the background signal is therefore unlikely to make a significant contribution to signal saturation effects, which is arguably the primary concern related to the contrast residue from a previous injection. Higher contrast dosages may require longer delays before a repeat injection.

**Figure 3:**
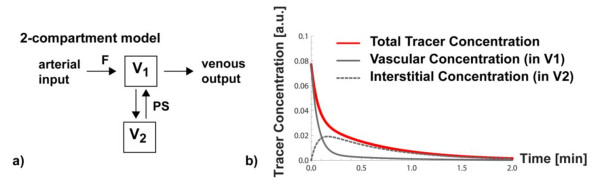
**The lumped two-compartment system**. a) Schematic diagram of lumped two-compartment model, where it is assumed that contrast is well-mixed within each compartment at any instant. Blood with tracer or contrast can flow at a rate F into a compartment with volume fraction v1 and exit on the venous side. The second compartment with volume v2, could represent the interstitial space, and the exchange of contrast between the two compartments is controlled by a parameter PS, representing the permeability surface area product for trans-capillary transport of contrast. b) The lumped two compartment system can be represented by a system of coupled linear differential equations (see appendix A). A solution for a unit impulse of contrast at t = 0, and assuming that there is no contrast in the two two compartments before this arterial input, was calculated for F = 0.88 ml/min/g and PS = 0.3 ml/min/g, assuming v1 = 0.06 ml/g and v2 = 0.18 ml/g. The red line represents the contrast concentration that would be detected per unit voxel volume, and which is calculated as volume-fraction-weighted sum of contributions from the vascular space (solid gray line) and the interstitial space (dashed gray line).

An important question is whether one wants to treat the vascular space as spatially lumped compartment ("well-stirred tank") with a uniform concentration of contrast, or whether one accounts for the fact that a vascular element has a spatial extent and the concentration of tracer can be higher at the arterial inlet(s) than further downstream. The latter approach results in a spatially dependent concentration of tracer or contrast. Mathematically this translates into the introduction of (a) spatial variable(s) into the set of (differential) equations that describe the tissue model, in addition to the time variable *t*, that describes the variation of contrast concentration with time. The compartmental model without spatial variable(s) only considers the change of contrast concentration as a function of time, and is termed a lumped compartment model. A prototypical example of a lumped two-compartment model is shown in Figure 3. The neglect of the spatial concentration gradients can result in an underestimation of the compartmental volumes, which can be verified by simulations where all parameters except the length of an axially-distributed blood-tissue exchange unit are kept constant. If the total distribution volume, including the vascular volume, is kept fixed for fitting of myocardial signal intensity curves to a model, then a lumped compartment model will result in an overestimate of blood flow, compared to a spatially distributed model. Using a spatially distributed model instead of a spatially lumped model can therefore have a significant positive impact on the accuracy of blood flow estimation.

For an extracellular contrast agent an important contribution to the total observed or measured tissue contrast concentration comes from the fraction of contrast that has traversed the capillary barrier and leaked into the interstitial space[35]. On signal intensity curves from myocardial perfusion studies the difference becomes clearly visible in the form of elevated signal intensity after the first pass of the contrast: contrast has passed into the interstitial space, and although it will eventually return to the vascular space and be washed out of the tissue region, the overall effect is a delay of the wash-out of contrast from the tissue region. For the extracellular contrast agent one needs to specify the volume of the interstitial space region, and also specify the rate at which contrast can traverse the capillary barrier, namely the permeability-surface area product (*PS*). Note, that it is not only the permeability of the capillary barrier that counts, but also the total area of this barrier, as a larger capillary surface area per unit tissue volume will result with constant permeability in a higher rate of contrast traversal from the vascular to the interstitial side. This means that capillary recruitment during vasodilation results in an increase of both blood flow and *PS *[36]. For an extracellular contrast agent one can assume that the capillary barrier passage is driven by the concentration difference between interstitial and extracellular spaces, and the *PS *rate constant is the same for either direction of transit. If the primary goal of the analysis is the quantification of blood flow, the other model parameters may appear as a nuisance. The focus on blood flow often captures only a very limited view of pathological changes, and effects like limited capillary recruitment, and a blunted vasodilator capacity can have a significant effect on *PS *and vascular volumes. Unfortunately, it is challenging to quantifying the permeability surface area product from first pass studies. A reliable estimate of *PS *may only be feasible with two contrast injections, using intravascular and extracellular contrast agents respectively.

Identifiability of a model parameter refers to the ability to measure or detect changes of the model parameter. For example, it turns out that with an extracellular contrast agent, the signal intensity curves measured with a CMR perfusion scan are relatively insensitive to the changes in vascular volume, because of the leakage of contrast into the interstitial space - the detected contrast enhancement corresponds to the effect of contrast in both the vascular and interstitial spaces. This means that signal intensity after the first pass primarily reflects the sum of the vascular and interstitial volume fractions, rather than just the vascular volume fraction. In the context of a "first pass" perfusion study, blood flow is a parameter that has a readily identifiable effect on the signal intensity curves, and this explains in part the reasons why "first pass" perfusion imaging, independent of modality, has mostly focused on the quantification of blood flow. For a model-based analysis of myocardial perfusion with an extracellular contrast agent this means that the vascular and interstitial volume parameters generally have to be kept at fixed assumed values, possibly with the constraint that their sum matches the effective distribution volume.

## Central Volume Principle

Model-independent analysis means that one foregoes specifying a functional model of the tissue structure. Model-independent analysis is based on the central volume principle introduced by Kenneth L Zierler in the the 1960's[37]. A 2002 review by Zierler offers an interesting historical retrospective on its conceptual development and experimental validation [38]. For its derivation one can start with an observation due to Eugen Fick, that the rate at which a substance accumulates in a tissue region of interest is given by the difference of concentrations of tracer substance flowing into and leaving the region, multiplied by the flow rate (F): *F*·(*c*_*out *_- *c*_*in*_) = *dq(t)/dt*, where *c*_*in, out *_denote the concentrations at (arterial) inlet and (venous) outlet, respectively, *q(t) *is the mass of tracer in the region, and *dq(t)/dt *its rate of change with time (*t*). Fick's principle is simply a statement of mass balance: tracer that has entered the region and not yet exited remains in the region of interest.

Starting from Fick's mass balance equation, one can arrive at an expression that relates the tracer amount in the region, *q(t)*, to its arterial input, in the form of a convolution integral, which according to Fick's principle (in integral form), also has to equal the amount of tracer that has entered the region, minus the amount that exited:

(3) $$q(t)=\int_{0}^{t}c_{in}(t-\tau )\cdot R_{F}(\tau )d\tau =F\int_{0}^{t}[c_{in}(\tau )-c_{out}(\tau )]d\tau$$

The function *R_F_(t) *represents the *q(t) *response if an impulse input of tracer is applied at the region input - this follows from the integral equation 3, if one replaces *c_in _*with a "Dirac-delta" input function. We also note, that with such an impulse input (*c_in _*(*t*) = *δ(t)) *at time t = 0, there can be no tracer at the output (*c_out _*(*t *= 0) = 0), as this would otherwise require that the tracer or contrast to traverse region instantaneously, i.e. *F → ∞*. It can be shown then, that *R_F_(t = 0) = F*, meaning the initial amplitude of the impulse response is equal to the blood flow through the region. This statement can be generalized to any type of arterial input and it represents the essence of the Central Volume Principle.

**Figure 4:**
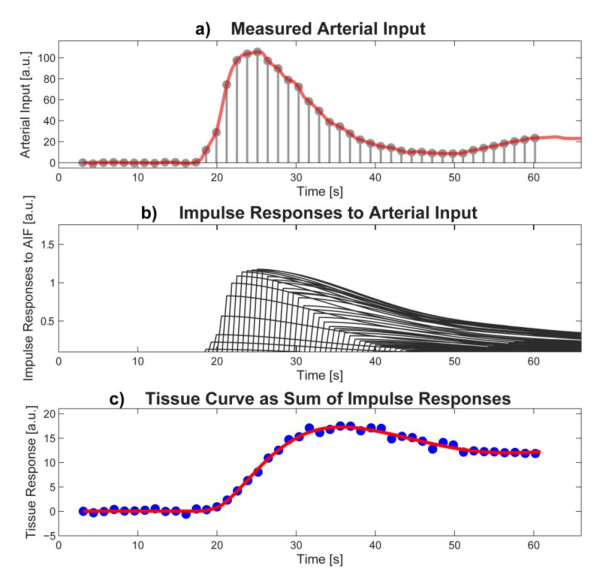
**Measured arterial input, impulse responses to arterial input and the tissue curve as a sum of impulse responses**. a) For an illustration of the Central Volume Principle it is useful to consider the arterial input as a sequence of impulses (gray lines with circle at top), whose amplitudes reproduce the measured arterial input (red line). b) Each of the impulses in the arterial input generates an impulse response in the tissue, which are all identical, except that each is scaled according to the amplitude of the corresponding impulse in the arterial input, and each impulse response is shifted so that its start coincides with the location of the arterial input pulse. c) The total tissue response can be calculated as the sum of the contributions from each impulse response. This sum is the numerical equivalent of the convolution integral of the arterial input with the impulse response. For deconvolution one attempts to reverse the above process and estimate from the signal curves in (a) and (b) the form of the impulse response.

The convolution integral in equation 3 allows one to calculate the *q(t) *response in a tissue region to a general from of arterial input *c_in_(t)*, which can include a recirculating component of the arterial input. The meaning of the convolution integral is illustrated in Figure 4. The Central Volume Principle allows one to quantify the blood flow through a region of interest, if the other quantities in equation 3, namely *q(t) *and *c_in_(t) *can be measured. The process of extracting *R_F_(t) *from the measurements of the ROI tracer concentration and arterial input concentration reverses the convolution operation, and is referred to as deconvolution, a mathematically substantially more challenging operation, than convolution.

The meaning of the impulse response can be further elucidated if interpreted as a probability. The value of *R_F_(t) *is normalized for this purpose by its value *R_F_(t = 0)*[39]. The normalized impulse response, *R(t) = R_F_(t)/R_F_(t = 0)*, gives for any time *t *the probability that a tracer molecule still remains in the region of interest at that time, assuming that it entered at *t = 0*. In other words, the normalized impulse response gives the probability that the tracer *residence *time is greater than *t*. The complement of this statement is that *1-R_F_(t)*, is the probability that the *transit *time of the tracer is <*t*. Such cumulative distribution functions for the transit times or residence times have to be monotonic functions, which can be a useful constraint for the estimation of an impulse response. From the cumulative distribution of transit times, one can obtain the probability density function (PDF) for transit times, *h(t)*, by taking the derivative of *1-R_F_(t)*.

A quantity often calculated from "first pass" perfusion studies is the mean transit time. It is defined as the first moment of the transit time PDF, *h(t)*. For myocardial perfusion studies with an intravascular contrast agent, one can use the tissue MTT and estimate of the vascular volume (*V*, e.g. from the ratio of steady-state signal intensities after contrast-enhancement in tissue and blood) to estimate blood flow:

(4) $$F=\frac{V}{MTT},$$

where MTT represents the MTT of tissue, after subtracting the MTT of the first pass of the AIF (i.e. by excluding the recirculation component). This corollary of the central volume principle for the special case of an intravascular tracer is frequently found in the literature on brain perfusion, because Gd-chelates are confined in the brain to the vascular bed by the blood brain barrier. For myocardial perfusion studies, equation 4 only applies when an intravascular contrast agent is used.

## Deconvolution Analysis

The Fourier convolution theorem states that convolution in the time domain is equivalent to point-wise multiplication of the Fourier transforms of the two quantities in the convolution integral. One could therefore think of deconvolution in the context of equation 3 as akin to point-wise division of $\hat{q}(\omega )$, the Fourier transform of *q(t)*, by ${\hat{c}}_{in}(\omega )$, the Fourier transform of the arterial input, to obtain the impulse response ${\hat{R}}_{F}(\omega )$ in the frequency domain. If ${\hat{c}}_{in}(\omega )$ is a relatively slowly varying function of time, it will have many locations at higher frequencies with close to zero, or zero amplitude. This would mean that point-wise division $\hat{q}(\omega )/{\hat{c}}_{in}(\omega )$ is a mathematically unstable approach for calculating the impulse response from the measured arterial and myocardial contrast enhancement. Although the described approach is useless in practice for deconvolution, the alluded to instability is symptomatic of the difficulties in performing a deconvolution.

Numerous approaches have been devised to address the deconvolution problem. Within the context of tracer-kinetic analysis, Axel introduced a useful parametric representation of the impulse response for the analysis of brain perfusion studies by computed tomography[40]. The particular parametric representation of the tissue impulse response introduced by Axel is known as Fermi function, and was chosen based on the insight that its shape resembles the expected shape of an impulse response for an intravascular tracer. Its mathematical representation is:

$$R_{F}(t)=\frac{A}{[\exp[(t-\mu )/k]+1}.$$

The parameter *t *represents time, and the parameters *μ*, *k*, and *A*, do not have a physiological interpretation, and should be viewed simply as "shape" parameters. For example, *μ *defines the width of the initial plateau, before the function decays at a rate set by the parameter *k*. Only the amplitude of *R_F_(t) *for *t = 0 *has a physiological meaning: it corresponds to the blood flow, according to the Central Volume Theorem. The "shape" parameters of the Fermi function can be determined with a non-linear least squares fitting algorithm, such as the Marquardt Levenberg algorithm. The fitting function is in this case given by the convolution of the Fermi-function with the arterial input. In an environment such as Matlab (The Mathworks, Natick, MA) it is straightforward to implement this method with a few lines of code.

**Figure 5:**
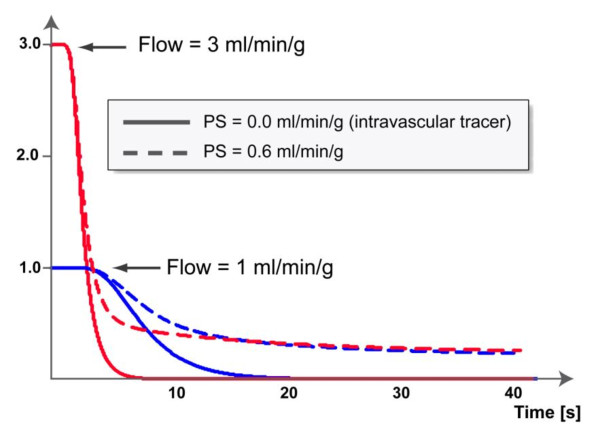
**Examples of impulse responses for intravascular and extracellular contrast agents**. Simulated impulse responses for a spatially distributed model, with myocardial blood flows of 1 (in blue) and 3 ml/min/g (in red), and for each flow level illustrating the differences between intravascular (solid line) and extracellular contrast agent (dashed line). In the latter case the permeability surface area product was assumed to be 0.6 ml/min/g. Of note is the initial plateau of the impulse response, which corresponds to the phase where no detectable amount of tracer has yet left the blood tissue exchange unit. For the case of an intravascular tracer, the initial plateau, followed by an exponential decay can be reproduced well with a Fermi-function as parametric representation of the impulse response.

**Figure 6:**
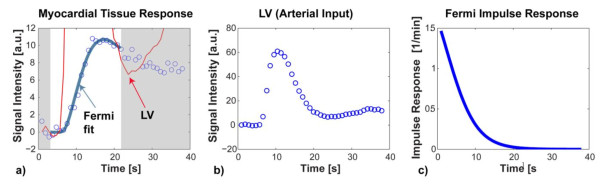
**Deconvolution of the measured myocardial signal intensity curve (blue circles) with the arterial input (b), was performed with a Fermi function model of the impulse response (c)**. Deconvolution of the measured myocardial signal intensity curve (blue circles) with the arterial input shown in (b), was performed with a Fermi function model of the impulse response as in (c). The time window for the fit excluded the portion of the signal intensity curve after the first pass in the blood pool (grayed-out). Also the initial transition of the myocardial signal to a steady state level, before injection of contrast was excluded (grayed out). The best fit to the measured data, shown as solid blue line in (a) was obtained by non-linear least-squares fitting, using a Marquardt-Levenberg algorithm in the Matlab environment (Mathworks, Natick, MA). The fitting function is calculated by convolution of the Fermi-function with the measured arterial input. The impulse response shape corresponding to this best fit is shown in panel (c). The myocardial blood flow is estimated from the initial amplitude of the Fermi impulse response.

It is noteworthy that the Fermi function can initially have approximately constant amplitude before decaying at an approximately exponential rate. This early plateau phase means that no, or a negligible amount of tracer has left the region of interest up to the end of the plateau duration, and such a plateau is more likely to be observed for low flows. This can be contrasted with the shape of an impulse response from a lumped two compartment model, which consists of a sum of two exponentials that begin to decay immediately for *t *>*0*. The difference is to be expected for a lumped compartment model, because, by definition, the contrast agent or tracer is well mixed at any moment within each compartment. The Fermi function represents a good approximation to the shape of impulse responses of intravascular tracers obtained by simulations from spatially distributed models. The limitation of the Fermi-function is the fact that it basically decays like a single exponential, while the most commonly used (extracellular) CMR contrast agent also permeates the interstitial space. For extracellular contrast agents the impulse response shows an initial fast decay, related to the vascular transit of contrast, followed by a slower, long-tailed decay that can be described as the delayed wash-out of contrast that has crossed the capillary barrier into the interstitial space. Examples of impulse responses for intravascular and extracellular contrast agents are shown in Figure 5. Still, the Fermi-function representation of the impulse response can be put to good use to estimate myocardial blood flow, by choosing for the analysis a window that does not exceed the first pass in the blood pool. During this early phase of contrast enhancement, any differences between intravascular and extracellular contrast agents are not readily noticeable, and the analysis provides a good estimate of myocardial blood flow. The shortest window for analysis of the contrast enhancement needs to encompass first pass in the blood pool. Numerical simulations have shown that this yields accurate estimates of the blood flow. In practice we set the end point to be at the signal intensity minimum between first pass and recirculation peaks in the blood pool. If the time window is extended, then this results in an increasing bias to underestimate the blood flow. An example is shown in Figure 6.

The representation of the impulse response shape can be generalized to properly reproduce also the features of the impulse response related to the delayed wash-out of contrast that has crossed the capillary barrier into the interstitial space. To that end, one can for example use a representation of the impulse response as a sum of B-spline components[41]. Nevertheless the algorithmic procedure to calculate the impulse response with such a B-spline basis becomes more complicated, and similar results for flow estimates are still obtained with the Fermi-function representation, by the sleight of hand described above.

It lies in the nature of the deconvolution problem that there is no unique solution for the impulse response, which is partially why it is considered an "ill-posed" problem. In fact, the brute force approach of calculating the impulse response from the Fourier transforms, or other naive forms of numerical inversion of the deconvolution operation yield mathematically admissible solutions of the convolution equation, which nevertheless have to be dismissed as unstable, and physiologically unrealistic. Under most circumstances we are not much concerned about the exact details of the impulse response, but are primarily interested in estimating the initial impulse response amplitude, which, by virtue of Zierler's central volume theorem, provides an estimate of the blood flow. But there are some requirements one can impose on the impulse response, e.g. that it has to be a monotonically decaying function - contrast can only leave the region of interest after the initial arterial impulse input and is not replenished. Furthermore one can impose some smoothness constraints, as impulse responses don't have any sudden jumps in the decay from their initial amplitude. Such requirements can help to stabilize the deconvolution operation.

The fact that with the deconvolution analysis one does not need to specify the internal structure of the blood tissue exchange unit also identifies its main limitation. At least with an extracellular contrast agent, one can with the deconvolution analysis determine only the blood flow, but not other perfusion parameters such as the vascular volume or the capillary permeability surface area product. For the latter one has to have some model to identify these parameters, both in the sense of giving them a functional meaning within a model of the blood tissue exchange unit, and also in the sense of being able to determine stable values from measurements of myocardial contrast enhancement.

## Water Exchange

The signal intensity observed during a study of myocardial contrast enhancement originates from the proton magnetization, and it often suffices to assume that the T1 or R1 changes are proportional to the concentration of contrast agent. This model can become inadequate when contrast agent is confined to subspaces in the tissue, while water can move between the tissue spaces. In this latter case the T1 of water protons changes not only in the subspaces where contrast is introduced through injection into the blood stream, but also in spaces that can exchange proton magnetization with these contrast-permeated subspaces. If the water exchange is slow in comparison to the difference of the native relaxation rates (relaxation rates in the absence of exchange), then one can neglect the effects of water exchange ("no exchange limit"). At the other extreme water exchange is so rapid that it can sample the relaxation environments in the two spaces multiple times during the relaxation recovery ("fast exchange limit"). In this latter case the effective relaxation rate corresponds to a weighted average of the intrinsic relaxation rates, with weights determined by the volume fractions of the two spaces. An example of fast water exchange is the relaxation of water in blood, which can rapidly move between blood plasma and red blood cells, because the dwell time of water in the red blood cells is relatively short (1-10 ms). In practice this means that the relaxation rate of water in blood is well described by a single exponential recovery at a rate that corresponds to the plasma concentration of contrast, reduced by a factor of (*1-Hct*) where *Hct *represents the blood hematocrit. This follows from considering the effective T1 of blood in the fast exchange limit, which is given by the volume-weighted average of the *T_1 _*of water in the blood plasma, $T_{1}^{plasma}$, and the T1 of water within the erythrocytes.

(5) $$\frac{1}{T_{1}^{eff}}=(1-Hct)\cdot R_{1}^{plasma}+Hct\cdot R_{1}^{rbc}=R_{1}^{blood}([Gd]=0)+(1-Hct)\cdot r_{1}[Gd]$$

$R_{1}^{blood}([Gd]=0)$ represents the relaxation rate constant of blood without contrast, r_1 _is the relaxivity of the contrast agent, and [Gd] its concentration in plasma. With fast water exchange the concentration of [Gd] in plasma is effectively diluted by the (1-Hct) factor.

Within myocardial tissue spaces water exchange (e.g. across the transcytolemmal barrier) often occurs at a rate that falls neither into the fast or slow exchange regimes, and this makes the analysis more tedious, and a description of the formalism is beyond the scope of this review[42]. Suffice it to say that the water exchange necessitates an expansion of the tissue model from a two to a three space model, to include the intra-cellular space, even though the contrast agent itself is excluded from the latter.

The conditions of fast or slow exchange are also determined by effective relaxation rate, which is a function of the pulse sequence applied during read-out of the signal. Assuming that a spoiled gradient echo sequence is used for read-out with higher flip angle radio-frequency pulses, then the effect of water exchange is less noticeable because the radio-frequency pulses, if applied with short repetition times (TR), rapidly erase any "memory" of water exchange. A more formal proof and experimental validation of this can be found in the work of Donahue et al.[43]. More recently Li et al showed how the combination of short TR and higher flip angle renders the observed contrast enhancement relatively insensitive to the effects of water exchange in myocardial tissue[44]. For a relaxation recovery not disturbed by radio-frequency pulses one has to use the full water exchange formalism, as the rate of water exchange falls into a range intermediate between the "no-exchange" and fast-exchange limits.

## Practical Recommendations to Facilitate Quantification

To counterbalance the partially abstract presentation in the earlier sections of this review, it may be useful to include a summary of practical experiences, and possible recommendations for quantification of myocardial blood flow. Tools for quantifying perfusion are on the verge of appearing in cardiac analysis software packages, but they will for several reasons retain the flavor of a research tool, that requires technical expertise for its use. Offering these tools in cardiac image analysis software packages remains a challenge because of the close link between acquisition protocols, and post-processing algorithms. As an example: The work-flow in the software programs may be built on the assumption that signal intensity in the ventricular cavity or the proximal aorta can be used to represent the arterial input of contrast. To what degree that is true, remains under the control of the user. Furthermore, methods to correct for signal saturation may require exact knowledge of the protocol parameters, not all of which may be encoded in the headers of the DICOM images. The user needs to be acutely aware of the limitation and built-in assumptions of the quantification algorithms to be used. At this point one can at least formulate a set of minimal requirements and recommendations that should be met if a user wishes to pursue absolute quantification of myocardial blood flow:

a) The pulse sequence needs to be chosen such that it allows the recovery of an arterial input function. For example, any technique where the signal in the blood pool is suppressed or attenuated by intraventricular flow (echo-planar technique with spin-echo) would not be suited for the goal of blood flow quantification. Another example is the interleaved notched saturation preparation[45], which is slice selective, and therefore produces T1-weighting in the blood pool that is flow and slice-position dependent. Other advantages of the notched-saturation technique have led to it being offered as standard protocol for myocardial perfusion imaging on one vendor platform.

b) The contrast dosage should be sufficiently low to allow recovery of contrast concentration, either by using a special acquisition technique, such as the dual-echo technique, or through post-processing, using calibration curves. For the standard gadopentetate dimeglumine agent, this generally means a contrast dosage well below 0.1 mmol/kg. If the non-linearity of contrast-enhancement versus contrast concentration in the blood pool should not exceed more than 10-15%, then the contrast dosage may need to be < 0.03 mmol or less at 1.5 and 3T.

c) For temporal resolution a repetition time corresponding to 1-2 R-to-R intervals at rest, and 1 R-to-R interval during stress is strongly recommended. Poor temporal resolution will lead to unacceptably low accuracy for MBF quantification[34]. Spatial resolution affects the accuracy of MBF quantification through partial volume effects, but MBF quantification is more forgiving of lower spatial resolution than temporal resolution, if partial volume effects are avoided. For adults, an in-plane spatial resolution < 2.5 mm should be aimed at. Parallel imaging (SENSE, SMASH, GRAPPA) has become a nearly standard component of myocardial perfusion imaging, using moderate acceleration factors around 2. With higher acceleration, the reduction of signal-to-noise may rapidly become the limiting factor, unless one uses a sequence technique such as steady-state free precession. Practical experience with SSFP perfusion imaging has shown that its use should be limited to 1.5 T or lower field strengths, and probably only with low-dosage contrast bolus[46].

d) New acceleration techniques, such as k-t BLAST and k-t SENSE[47,48], allow perfusion imaging with high spatial resolution, but the algorithms applied for image reconstruction give rise to some degree of low-pass temporal filtering if high acceleration factors (~≥8) are used. Such low-pass temporal filtering, will lead to a bias to underestimate flow. The resolution of this problem will have to await further advances in these combined spatial-temporal acceleration techniques.

e) CMR at 3 Tesla is steadily gaining ground. For myocardial perfusion imaging with gradient echo techniques (without steady-state free precession) one can make an unreserved recommendation for the higher field strength, because of the concomitant increase of signal-to-noise compared to 1.5 Tesla. Although SSFP techniques yield excellent signal-to-noise ratio at 1.5 Tesla[17,18], there are well-founded concerns that contrast enhancement with SSFP can be modulated by confounding factors such as frequency shifts during bolus passage[46].

## Current Limitations

Arguably one of the most serious limitations of quantitative perfusion analysis is the inability to measure the arterial input accurately and relatively closer to the myocardial region of interest. This has a direct impact on the accuracy of blood flow estimates. Instead the enhancement from the contrast bolus is typically measured in the LV blood pool or the proximal aorta, but transit through the epicardial vessels is bound to cause some dispersion of the contrast bolus, which may be exacerbated by an epicardial stenosis[49]. One is forced to either neglect this dispersion of the contrast bolus during transit through the epicardial vessels, or assume a fixed relative dispersion. A neglect of the dispersion results in an underestimation of blood flow.

## Summary

Cardiac magnetic resonance can be used to quantify absolute myocardial blood flow with high spatial resolution, thereby avoiding spill-over of signal from the LV cavity, and providing an assessment of the transmural perfusion gradient. The quantification of blood flow is not significantly more time consuming that a semi-quantitative analysis, in particular if a deconvolution method is used for the analysis, requiring minimal user input. Instead the most time-demanding step continues to be the segmentation of the images along endo and epicardial borders. Standardization of the quantitative analysis still appears to be some time off, as this remains a field of active investigation, where further optimization and innovations are still forthcoming at a rate that precludes a broad consensus and standardization. Despite its evolving status, quantitative CMR perfusion imaging has achieved recognized role in studies of coronary physiology and cardiac diseases. Whether it will be adopted for the clinical workflow remains uncertain at this time.

## Appendix A

## Example of a two compartment model for analysis of myocardial perfusion

We consider as an example a spatially-lumped, two compartment model with flow, F, capillary volume V_1 _and interstitial volume V_2_, their respective concentrations, C_1 _and C_2_, and an exchange coefficient PS, which stands for the permeabilty-surface area product. The equations describing this model are:

(6) $$V_{1}\frac{dc_{1}(t)}{dt}=F\left(c_{aif}(t)-c_{1}(t)\right)+PS\left(c_{2}(t)-c_{1}(t)\right)$$

(7) $$V_{2}\frac{dc_{2}(t)}{dt}=PS\left(c_{1}(t)-c_{2}(t)\right)$$

Note that the exchange is passive, as it depends only on the difference in concentrations between the compartments. The tracer concentration in a tissue region described by this two-compartment model is given by weighted sum of *c_1_(t) *and *c_2_(t)*, with weights corresponding to the volume fractions of the two compartments:

$$c(t)=\frac{\nu_{1}c_{1}+\nu_{2}c_{2}}{\nu_{1}+\nu_{2}}.$$

It can be shown that the solution of the equations (1) and (2) to an impulse input is given by the sum of two exponential functions with coefficients that are proportional to the volumetric flow rate per unit volume. Obtaining the solution to this set of equations involves some tedious algebra which we spare the reader. Instead we included a Mathemetica notebook (Additional file 1), which can be opened with the Mathematica software package, or with the free Mathematica player software, which can be downloaded from http://www.wolfram.com.

This two-compartment model is used to illustrate the effects of changes in flow, PS, and compartmental volumes on the impulse response, and on the tissue residue curves. The tissue residue curves correspond to the signal intensity curves that are measured for myocardial regions of interest in a CMR perfusion study. We also verify that the amplitude of the impulse response for this lumped two-compartment model corresponds to the tissue blood flow, as predicted by Zierler's central volume theorem.

## Competing interests

The author declares that they have no competing interests.

## Supplementary Material

Additional file 1 **Two compartment model for analysis of myocardial perfusion**. The solution of the equations (1) and (2) to an impulse input is given by the sum of two exponential functions with coefficients that are proportional to the volumetric flow rate per unit volume. The additional file contains the algebra in a Mathemetica notebook used to obtain the solution to this set of equations. It can be opened with the Mathematica software package, or with the free Mathematica player software, which can be downloaded from http://www.wolfram.com. Click here for file
